# Exogenous Silicon Enhanced Salt Resistance by Maintaining K^+^/Na^+^ Homeostasis and Antioxidant Performance in Alfalfa Leaves

**DOI:** 10.3389/fpls.2020.01183

**Published:** 2020-08-26

**Authors:** Yuanfa Meng, Qiang Yin, Zhijian Yan, Yuqing Wang, Jianming Niu, Jie Zhang, Kai Fan

**Affiliations:** ^1^Institute of Grassland Research, Chinese Academy of Agricultural Sciences, Hohhot, China; ^2^School of Ecology and Environment, Inner Mongolia University, Hohhot, China

**Keywords:** silicon, alfalfa, salt tolerance, ion homeostasis, antioxidant, photosynthesis

## Abstract

Silicon (Si) has been known to enhance salt resistance in plants. In this experiment, 4-weeks-old alfalfa seedlings were exposed to different NaCl concentrations (0–200 mM) with or without 2 mM Si for two weeks. The results showed that NaCl-stressed alfalfa seedlings showed a decrease in growth performance, such as stem extension rate, predawn leaf water potential (LWP) and the chlorophyll content, potassium (K^+^) concentration, as well as the ratio of potassium/sodium ion (K^+^/Na^+^). In contrast, NaCl-stressed alfalfa seedlings increased leaf Na^+^ concentration and the malondialdehyde (MDA) level, as well as the activities of superoxide dismutase (SOD), catalase (CAT), and peroxidase (POD) in alfalfa leaves. Besides, exogenous Si application enhanced photosynthetic parameters of NaCl-stressed alfalfa seedlings, which was accompanied by the improvement in predawn LWP, level of chlorophyll content, and water use efficiency (WUE). The Si-treated plants enhanced salinity tolerance by limiting Na^+^ accumulation while maintaining K^+^ concentration in leaves. It also established K^+^/Na^+^ homeostasis by increasing K^+^/Na^+^ radio to protect the leaves from Na^+^ toxicity and thereby maintained higher chlorophyll retention. Simultaneously, Si-treated plants showed higher antioxidant activities and decreased MDA content under NaCl stress. Our study concluded that Si application enhanced salt tolerance of alfalfa through improving the leaves photosynthesis, enhancing antioxidant performance and maintaining K^+^/Na^+^ homeostasis in leaves. Our data further indicated exogenous Si application could be effectively manipulated for improving salt resistance of alfalfa grown in saline soil.

## Introduction

Soil salinization is a major abiotic stress that hinders plant growth and crop production in the world (Ahmad et al., 2019). Soil salinization has occurred in approximately 20% of irrigated land which accounts for one-third of land for food production (Shrivastava and Kumar, 2015; Gregory et al., 2018). Arid and semi-arid regions are particularly susceptible to soil salinization, where irrigation is employed routinely to promote crop yield. But this problem is predicted still worsen due to continued global warming and extreme climate fluctuations. Soil salinization reduces the land use value, causes huge crop yield losses, and threatens the ecological environment, thus presenting the economic and ecological hazards (Long et al., 2014).

Salinity stress adversely affects the plant morphological features and some important processes related to physiology and biochemistry (Ashraf, 2004). The harm of soil salinization to plants is typically divided into two phases. The first phase of salinity is the osmotic effect. The soil water potential was reduced with the increasing concentration of salts in the soil solution, which hinders the absorption of water from the roots, resulting in plant water deficit (Pardo, 2010; Roy et al., 2014). Plants respond to this water deficit signal rapidly from roots to shoots, resulting in the decrease of intracellular turgor and slow cell expansion (Munns, 2005; Munns and Tester, 2008). This response also activates abscisic acid (ABA) biosynthesis and reduces stomatal conductance (Munns, 2005; Munns and Tester, 2008; Roy et al., 2014), thus interfering with the photosynthesis. Water deficit will reduce the stomatal conductance, which decreases the carbon assimilation and biomass production (Almeida et al., 2017). The second phase of salinity is ionic toxic: leaves, especially old leaves, accumulate the Na^+^ to a toxic level, causing leaf chlorosis and senescence (Roy et al., 2014). At the same time, the accumulation of Na^+^ in photosynthetic tissue will reduce photosynthetic carbon assimilation (Munns et al., 2020), resulting in loss of yield (Roy et al., 2014; Rubio et al., 2020). This can also cause an ionic imbalance. On the one hand, Na^+^ competes with K^+^ for intracellular influx due to common protein transporters. On the other hand, some key metabolic processes were disturbed because Na^+^ interferes with K^+^ for major binding sites. Salt stress disturbs K^+^ homeostasis and harms plant performance. Both osmotic pressure and ionic effects destroy the cell’s metabolism, leading to excessive accumulation of reactive oxygen species (ROS), which adversely affects tissue structure and cell metabolism (Chaves and Oliveira, 2004; Chaves et al., 2009).

Alfalfa (*Medicago sativa* L.) is a perennial legume forage, which is an important fodder for intensive dairy farm production in northern regions of China. Due to its high nutritional value and symbiotic nitrogen fixation, alfalfa has become one of the most common cultivated varieties in the agricultural forage system. However, the alfalfa planting area is generally exposed to different salinity stress, which has a great impact on its growth performance. It has been known that most alfalfa cultivars are relatively sensitive to salt stress; the high concentration of salts in the soil causes poor emergence and the failure establishment of alfalfa seedlings (Al-Niemi et al., 1992). Alfalfa salinity research mainly focuses on the germination and seedling establishment periods (Wang et al., 2009). For example, studies have shown the emergence rate of alfalfa begins to decrease when the soil salinity is above 22 mM NaCl (Anower et al., 2013; Campanelli et al., 2013). Moreover, it is approximately a 7.3% reduction of alfalfa production for each dS m^−1^ when the soil salinity exceeds 2.0 dS m^−1^ (Maas and Hoffman, 1977; Johnson et al., 1992). Hence, it is extremely important to develop new strategies to enhance salt tolerance of alfalfa seedlings in saline soils, and thereby promote alfalfa productivity.

Silicon (Si) is among the abundant elements in the earth’s crust (Epstein, 1999) and has shown various benefits to plant performance (Ma and Yamaji, 2006). An increased plant-available Si can promote plant growth after Si application (Li et al., 2019; Li and Delvaux, 2019), especially under stress conditions (Zhu and Gong, 2014). This increased plant-available Si improved the salt resistance of wheat and decreased the inhibition of Na^+^ toxicity and the oxidative stress damage as wheat matures, especially at the booting stage (Daoud et al., 2018). Exogenous Si can improve the salt resistance in different Si accumulator plants, such as rice that belongs to Si active accumulator, cucumber that has higher Si accumulation in the shoots, tomato that has low content of Si in the tissue (Mitani and Ma, 2005). Currently, studies have shown that exogenous Si application can improve plant salt resistance (Li et al., 2015; Liu et al., 2015a). Exogenous Si can reduce the content of MDA under salt stress (Liang et al., 2003; Moussa, 2006; Soylemezoglu et al., 2009). Studies have also demonstrated that Si can improve some antioxidant enzymes’ performance such as superoxide dismutase (SOD), peroxidase (POD), and catalase (CAT) under saline conditions. The decrease of MDA content and the performance of antioxidant enzymes for exogenous Si-supplied plants may be the reason for the improved salt resistance. Besides, Liang et al. (2006) showed that Si activates some H+-ATPases related to Na^+^ and K^+^ transport, which are considered to maintain ion homeostasis under salt stress.

Many recent works have expanded our understanding of Si-induced alleviation of salt stress in many crop varieties. However, the understanding of Si-improved salt resistance mechanisms in alfalfa is still relatively limited. Besides, the mechanisms of Si-induced alleviation of salinity may be different among species, which should be demonstrated for the salt resistance of alfalfa. Thus, in our study, we tried to explore the feasibility of Si amendments in alfalfa seedlings under NaCl-stressed through plant growth feature measurements. Further, a combination of chlorophyll concentration, predawn leaf water potential, tissue ion concentration, ROS content and lipid peroxidation, photosynthesis parameters, and antioxidant enzymes were carried out in alfalfa leaves to elucidate the mechanisms related to Si-induced salt resistance.

## Materials and Methods

### Plant Material and Growth Conditions

The pot experiment was performed at Ordos key research station for field observation of ecological environment on sandy grassland, Ministry of Agriculture and Rural Affairs, China (N40°26′ E109°58′). This region belongs to a temperate continental climate with a mean annual rainfall of 310 mm, a humidity index of 0.4, a mean annual temperature of 6°C, 156 frost-free days, and annual sunshine of 3,000 h. The soil was sampled from the field of 0^_^20 cm in the Ordos forage base. The main chemical properties of soil were characterized as follows: pH 7.84, soil organic matter 10.41g kg^−1^, available N 110.32 mg kg^−1^, available P 121.16 mg kg^−1^, available K 64.55 mg kg^−1^, and available Si 85.59 mg kg^−1^. Commercial alfalfa (cv. concept) was used in the experiment, which has a better adaptability and higher yield to the local sandy soil in our previous three-year (2012–2014) test results.

### Experimental Design and Treatments

The study was performed in a two factorial completely randomized block design with four replications. The factors comprised (i) Si level (0 and 2 mM), (ii) NaCl level (0, 50, 100, and 200 mM). The control is 0 mM NaCl without Si supplement. Each treatment consists of four pots, 32 pots for all treatments. The sowing date was May 10^th^. Alfalfa seeds (*M. Sativa* L. cv. concept) were surface-sterilized with mercuric chloride (0.1%) for 5 min and rinsed three times with distilled water, and then air-dried. Uniform seeds were chosen and sown in pots (ten seeds/pot) with 1.5 kg of air-dried soil. After the emergence of the first trifoliate leaf (10 days after sowing), four alfalfa seedlings were kept in each pot, and the pot was irrigated to maintain at field capacity with tap water. Si as Na_2_SiO_3_·5H_2_O was dissolved in water, and sodium silicate solution was passed through a column with cation-exchange resin to remove Na^+^ ions below 10^−2^ mM Na (Li et al., 2019). Salt treatments and Si were conducted simultaneously 4 weeks after sowing and continued for 2 weeks. To avoid osmotic shock, the solutions were divided into four times (250 ml/per time) to irrigate the alfalfa seedlings in four days, with each pot receiving 1,000 ml of the treated solutions. Leaching was avoided at each pot. The pots were watered with tap water at field capacity during the treatment (100 ml/three days).

### Measurement of Morphological Features and Chlorophyll Concentration

The whole plant was sampled after treatment for 14 days. One plant was randomly chosen from each pot. Each treatment has four replicates. The roots and shoots were separated manually and then dried at 80°C in an oven for 48 h, and the dry weights were recorded. The stem extension rate was calculated by recording the length of the main stem of each plant before and after the treatment. Total chlorophyll (Chl) content was determined using the method of Arnon (1949) with some modifications. We collected five leaves randomly from each pot. Sample of 0.2 g leaf matter was ground to powder and extracted with 10 ml of 80% acetone (v/v). Then the extract was centrifuged at 4,000×g at 4°C for 10 min, and the supernatant was used for spectrophotometer readings. The absorbance of the supernatant was determined at 645 and 663 nm by a UV spectrophotometer. Total chlorophyll was calculated using the following formula: Total chlorophyll = 80.2A_663_ + 20.2A_645_. The content of the chlorophyll was expressed as milligram per gram FW.

### Determination of Predawn Leaf Water Potential

The predawn LWP was determined by Dewpoint Potential Meter (WP4-T, Decagon Devices, Inc., Pullman, USA) between 6:00 and 6:30 a.m. after 14 days of treatment. Ten fully expanded young leaves were randomly excised from each pot. First, a drop of distilled water was applied to the leaf surface, then the leaf surface was abraded gently and evenly with a piece of 600-grit sandpaper. Dry the leaf surface thoroughly with a lint-free tissue. Immediately seal leaves with a moist towel to a plastic bag. Place the samples into the WP4-T sample cup and cover the bottom to measure leaf water potential.

### Determination of Active Oxygen Species and Lipid Peroxidation

O_2_^•−^ was estimated with some modifications of the method of Elstner and Heupel (1976) by monitoring the nitrite formation from hydroxylamine in the presence of O_2_^•−^. Ten fully expanded young leaves were randomly excised from each pot. Each treatment included four replications. Sample of 0.5 g frozen leaf matter was added 3 ml of 65 mM potassium phosphate buffer (pH 7.8) and centrifuged at 5,000×g for 10 min. The supernatant (1 ml) was added 0.9 ml of 65 mM phosphate buffer (pH 7.8) and 0.1 ml 10 mM hydroxylamine hydrochloride, then the mixture was incubated at 25°C for 20 min. The incubation mixture was added 17 mM sulphanilamide and 7 mM *α*-naphthylamine and reacted at 25°C for 20 min; 2 ml of ethyl ether was added to the reaction mixture and centrifuged at 1,500×g for 5 min. The absorbance in the aqueous solution was read at 530 nm. A standard curve with NO_2_^−^ was used to calculate the production rate of O2^•−^ from the chemical reaction of O2^•−^ and hydroxylamine.

For the estimation of H_2_O_2_ content, the method of Velikova et al. (2000) was used. Ten fully expanded young leaves were randomly excised from each pot. Each treatment included four replications. Sample of 0.2 g fully expanded young leaves was homogenized in 0.1% trichloroacetic acid (TCA) and centrifuged at 12,000×g. 10 mM potassium phosphate buffer (pH 7.0) (0.5 ml) and 1 M KI solution (1 ml) were added to the leaf extract solution (0.5 ml). The absorbance of the reaction mixture was measured at 390 nm. The content of H_2_O_2_ was determined by using a standard curve prepared with a graded solution of H_2_O_2_.

Lipid peroxidation was measured as the content of MDA that was determined by the thiobarbituric acid (TBA) reaction based on the method described by Heath and Packer (1968) with some modifications. Ten fully expanded young leaves were randomly excised from each pot. Each treatment included four replications. Sample of 0.5 g fully expanded young leaves was homogenized in 5.0 ml of 5% (w/v) TCA and centrifuged at 12,000×g for 10 min. The extract (2 ml) was mixed with 0.6% TBA (2 ml) and put in a boiling water bath for 10 min. Then the absorbances of the reaction mixture were measured at 532 and 600 nm. The concentration of MDA was calculated based on the difference in absorbance (A_532_ − A_600_) and was expressed as micromole per gram FW by using the MDA extinction coefficient of 155 mM^−1^ cm^−1^.

### Measurements of Leaf Gas Exchange Parameters and Leaf Water Potential

At 9:00–11:00 a.m. on the 14^th^ day after treatment, upper fully expanded young leaves were selected for the measurement of the transpiration rate (*Tr*), stomatal conductance (*gs*), and photosynthesis rate (*Pn*) using the LI-6400XT portable photosynthesis system (Li-Cor, Inc., Lincoln, NE, USA). We selected three leaves per plant in each pot to measure four reputations of each parameter. Each treatment included four replications. Leaves were exposed to saturating irradiance of 1,200 μmol m^−2^ s^−1^ with an LI-6400 red/blue LED light. The flow rate was 300 μmol m^−2^ s^−1^, and the leaf temperature was 28°C (Liu et al., 2015b). At the leaf level, the instantaneous water use efficiency (WUE, mmol CO_2_ mol^−1^ H_2_O) was estimated as WUE = A/E, where A is photosynthesis rate and E is transpiration rate (Ahmad et al., 2013).

### Assays for Antioxidant Enzyme Activities

Samples were extracted from the fully expanded young leaves and determined by Mukherjee and Choudhuri (1983) with some modifications. Ten expanded young leaves were randomly chosen from each pot. There were four replications in each treatment. Sample of 0.3 g fresh matter was ground with 0.1 mM potassium phosphate buffer (PBS) (pH 7.8) on ice by using a mortar into a homogenate. The mixture was centrifuged at 10,000×g at 4°C for 20 min, then the supernatant was stored at 4°C for the measurement of the antioxidant enzyme activity.

SOD (EC 1.15.1.1) activity was measured by using the nitro blue tetrazolium method (Giannopolitis and Ries, 1977). The enzyme extract (0.1 ml) was added 100 mM phosphate buffer (pH7.6), 1.5 mM Na_2_CO_3_, 2.25 mM NBT, 200 mM methionine, 3 mM ethylene diamine tetraacetic acid (EDTA), 0.06 mM riboflavin, and distilled water. The reaction tubes with enzyme extract were illuminated with a 15 W fluorescent lamp for 10 min. The other set of tubes without enzymes served as control and illuminated at the same conditions. The complete reaction mixtures without illumination were used as blank. The absorbance of the reaction mixture was measured at 560 nm with a UV spectrophotometer (UV-2700, Shimadzu, Kyoto, Japan). One unit of SOD activity was expressed as the amount of enzyme that was required to inhibit the rate of NBT reduction by 50% in the controls without enzymes.

Peroxidase (EC 1.11.1.7) activity was measured by using the method described by Maehly and Chance (1954). The enzyme extract (0.1 ml) was added acetate buffer (0.1 mol/L^−1^, pH 5.4), ortho-dianisidine (0.25% in ethyl alcohol) and 0.1 ml 0.75% H_2_O_2_ solution. The absorbance change of the brown guaiacol at 460 nm was measured for determining the POD activity. The enzyme activity level was expressed as the quantity of guaiacol oxidized by POD per minute.

CAT (EC 1.11.1.6) activity was measured using the method described by Aebi (1984). The reaction mixture (3 ml) contained 50 mmol/L^−1^ potassium phosphate buffer (pH 7.0), 10 mmol/L^−1^ H_2_O_2_ solution. The reaction mixture was initiated by adding 200 µl of the enzyme extract and the activity was estimated by measuring the initial rate of disappearance of H_2_O_2_ at 240 nm (*E* = 39.4 mM^−1^ cm^−1^) for 30 s. One unit of CAT activity was calculated as a 0.01 unit min^−1^ change in absorbance.

### Tissue Ion Concentrations Measurement

Ten expanded young leaves were randomly chosen from each pot. There were four replicates for each treatment. Then leaves were dried in an oven at 80°C for 48 h and milled to a powder. Samples of 0.2 g leaves matter were acidified with HNO_3_ for 12 h and then digested using a microwave digestion system for elemental analysis. Elemental concentrations were measured with an inductively coupled plasma optical emission spectrometer (ICP-MS, Agilent 7700 ×, Agilent Technologies, USA) (Zhao et al., 2018).

### Determination of Si Concentration

For the measurement of Si concentration, the method of Kabir et al. (2016) was adopted with some modifications. First, fully expanded young leaves were washed with CaSO_4_ and deionized water thoroughly, then the leaves were ground in mortar-pestle. The sample was centrifuged at 12,000×g for 5 min, and the supernatant was then transferred to 10% ammonium molybdate. Afterward, the supernatant mixture was added 10% oxalic acid to form a silico-molybdate complex, then 0.5% ascorbic acid was added to the reaction mixture and kept at 28°C for 30 min until the blue color was formed. The absorbance of the reaction mixture was then recorded at 660 nm in a UV spectrophotometer (UV-2700, Shimadzu, Kyoto, Japan). There were four replicates for each treatment.

### Statistical Analyses

The results were analyzed using SPSS 20.0. The variations in data were expressed as means ± standard deviation (SD) of four independent replicates of each treatment. Data were analyzed at the significance level of *P* < 0.05 using the ANOVA two-way and Duncan’s multiple range tests. The graphs were drawn using Origin software v.8.5.

## Results

### Effect of Si on Alfalfa Growth Features During Salt Exposure

The shoot DW, root DW, and stem extension rate significantly decreased as the NaCl concentration increased (**Tables 1** and **2**). However, shoot DW significantly increased due to Si supplementation under each NaCl treatment, especially at the 100 mM NaCl treatment, at which the maximum recovery was observed (**Tables 1** and **2**). The root DW and stem extension rate significantly increased at 100 mM NaCl treatment when adding Si. Si supplementation alone significantly increased the shoot dry weight, while it did not significantly affect the root DW and stem extension rate compared with control.

**Table 1:** Morpho-physiological features of alfalfa after exposure to 0, 50, 100, and 200 mM NaCl with (+2 mM Si) or without (−2 mM Si) Silicon for 14 d.

Treatment NaCl (mM)	Si(2 mM)	Shoot DW (g·plant^−1^)	Reduction(%)	Root DW(g·plant^−1^)	Reduction(%)	Stem extension rate(mm·d^−1^)	Reduction(%)
0	−Si	15.53 ± 0.72b	–	4.83 ± 0.65ab	–	15.54 ± 0.68a	–
	+Si	16.18 ± 0.19a	–	5.14 ± 0.12a	–	15.71 ± 0.58a	–
50	−Si	13.34 ± 0.22d	14.12	4.50 ± 0.19b	6.83	11.43 ± 0.82bc	26.5
	+Si	14.40 ± 0.38c	7.28	4.60 ± 0.22b	4.71	12.32 ± 0.90b	20.7
100	−Si	9.93 ± 0.39e	36.05	3.93 ± 0.26c	18.74	9.64 ± 0.92d	37.9
	+Si	12.96 ± 0.42d	16.53	4.27 ± 0.08b	11.5	11.07 ± 0.41c	28.6
200	−Si	6.20 ± 0.30g	60.08	2.51 ± 0.22d	48.03	6.79 ± 0.92e	56.3
	+Si	7.41 ± 0.39f	52.29	2.83 ± 0.06d	41.51	7.32 ± 0.68e	52.9

Values are expressed as mean ± SD. The diﬀerent letters indicate signiﬁcant diﬀerences between treatments (p < 0.05). DW, dry weight.

**Table 2:** Two factor ANOVA (Si and NaCl stress) for all parameters studied of alfalfa significance values.

	Si application (Si)	NaCl stress (S)	Interaction (Si × S)
Shoot DW	<0.001	<0.001	<0.05
Root DW	<0.05	<0.001	<0.05
Stem extension rate	<0.001	<0.001	<0.05
Total chlorophyll	<0.001	<0.001	<0.05
Predawn LMP	<0.001	<0.001	<0.001
Si content	<0.001	<0.001	<0.001
Na^+^ content	<0.001	<0.001	<0.05
K^+^ content	<0.001	<0.001	<0.05
Ratio of K^+^/Na^+^	<0.001	<0.001	<0.05
MDA content	<0.001	<0.001	<0.001
H_2_O_2_ content	<0.001	<0.001	<0.001
O_2_^•−^ content	<0.001	<0.001	<0.001
Pn	<0.001	<0.001	<0.05
Tr	<0.001	<0.001	<0.05
*Gs*	<0.001	<0.001	<0.001
WUE	<0.05	<0.001	<0.05
SOD	<0.001	<0.001	<0.05
POD	<0.001	<0.001	<0.05

### Effect of Si on the Contents of Photosynthetic Pigments During Salt Exposure

**Figure 1:**
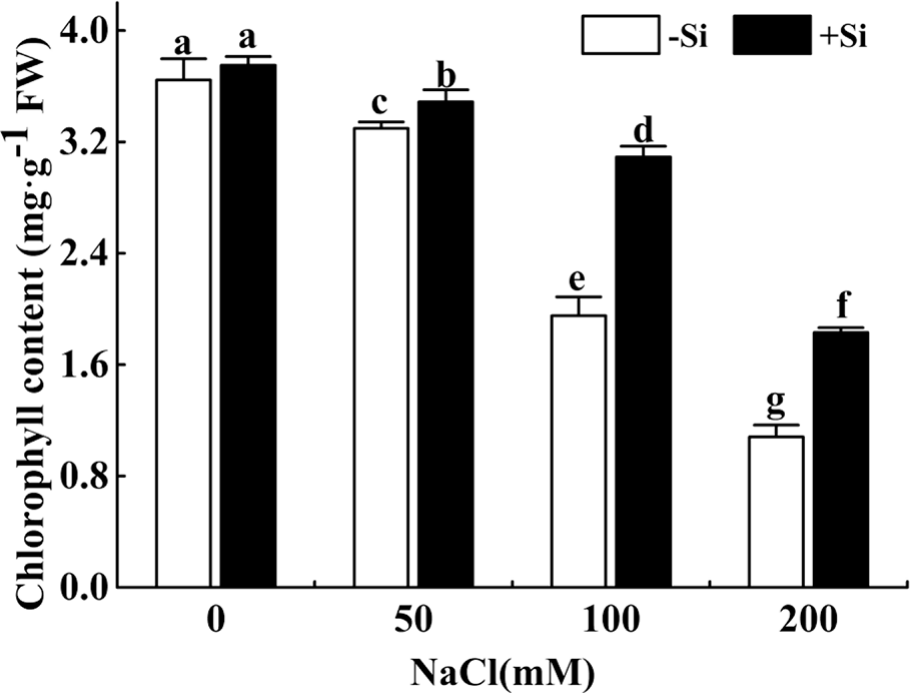
The effect of salt and Si treatment on the leaf chlorophyll content in alfalfa. Four weeks-old alfalfa seedlings were exposed to 0, 50, 100, and 200 mM NaCl with (+2 mM Si) or without (−2 mM Si) Silicon for 14 d. Leaves were sampled 14 d after conducting salt and Si treatment to measure chlorophyll concentration. Bars indicate standard deviation (SD) of the means (*n* = 4). Different letters denote signiﬁcant differences among the treatments based on Duncan’s multiple range test (*P* < 0.05). FW, fresh weight.

The total chlorophyll concentration showed a significant decrease with the rise of NaCl concentration from 0 to 200 mM (**Figure 1**). However, the total chlorophyll concentration significantly increased due to Si supplementation under the NaCl concentration from 50 to 200 mM.

### Effect of Si on the Water Status in Leaves During Salt Stress

**Figure 2:**
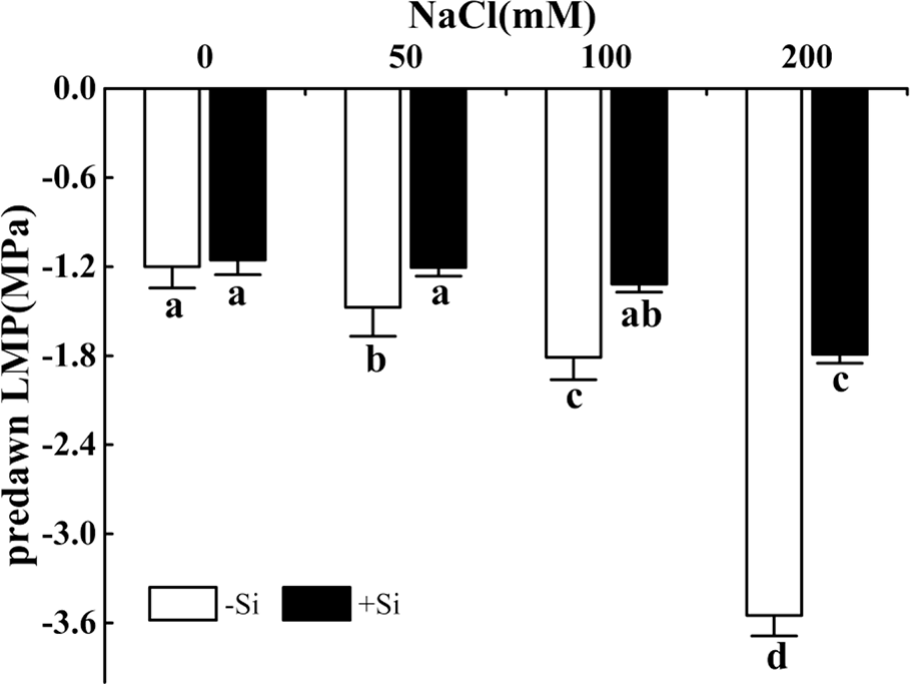
The effect of salt and Si treatment on predawn LWP in alfalfa leaves. Four weeks-old alfalfa seedlings were exposed to 0, 50, 100, and 200 mM NaCl with (+2 mM Si) or without (−2 mM Si) Silicon for 14 d. Leaves were measured 14 d after conducting salt and Si treatment to determine predawn LWP. Bars indicate standard deviation (SD) of the means (*n* = 4). Different letters denote signiﬁcant differences among the treatments based on Duncan’s multiple range test (*P* < 0.05). predawn LWP, predawn leaf water potential.

The predawn LWP significantly decreased as the NaCl concentration increased from 0 to 200 mM (**Figure 2**). However, the predawn LWP significantly increased due to Si supplementation under the NaCl concentration from 50 to 200 mM. Leaves of plants grown with Si alone showed similar predawn LWP values compared with control (**Figure 2**).

### Effect of Si on Si Content and K^+^/Na^+^ Homeostasis in Alfalfa Leaves During Salt Stress

**Figure 3:**
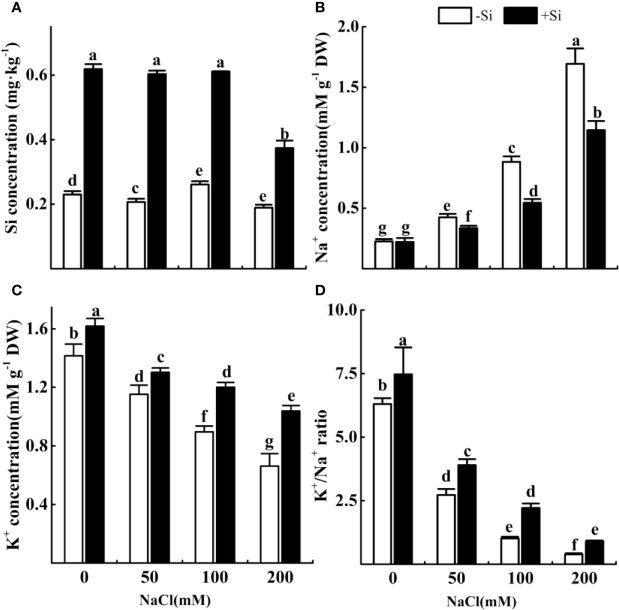
The effect of salt and Si treatment on the Si content **(A)**, Na^+^ concentrations **(B)**, K^+^ concentrations **(C)** and K^+^/Na^+^ ratio **(D)** in alfalfa leaves. Four weeks-old alfalfa seedlings were exposed to 0, 50, 100, and 200 mM with (+2 mM Si) or without (−2 mM Si) Silicon for 14d. Leaves were sampled 14 d after conducting salt and Si treatment to determine the ion concentrations. Bars indicate standard deviation (SD) of the means (*n* = 4). Different letters denote signiﬁcant differences among the treatments based on Duncan’s multiple range test (*P* < 0.05). DW, dry weight.

The Si content in leaves significantly increased when adding Si in all treatments (**Figure 3A**). The concentration of Na^+^ significantly increased with the rise of NaCl concentration from 0 to 200 mM (**Figure 3B**). However, the content of Na^+^ significantly decreased due to Si supplementation under the NaCl concentration from 50 to 200 mM. The concentration of K^+^ significantly decreased with the increasing NaCl concentration (**Figure 3C**). However, the concentration of K^+^ significantly increased due to Si supplementation with each NaCl treatment. The ratio of K^+^/Na^+^ significantly decreased under the NaCl concentration from 0 to 200 mM (**Figure 3D**). However, the ratio of K^+^/Na^+^ significantly increased due to Si supplementation with each NaCl treatment.

### Effect of Si on the NaCl-Induced Oxidative Damage in Leaves During Salt Exposure

**Figure 4:**
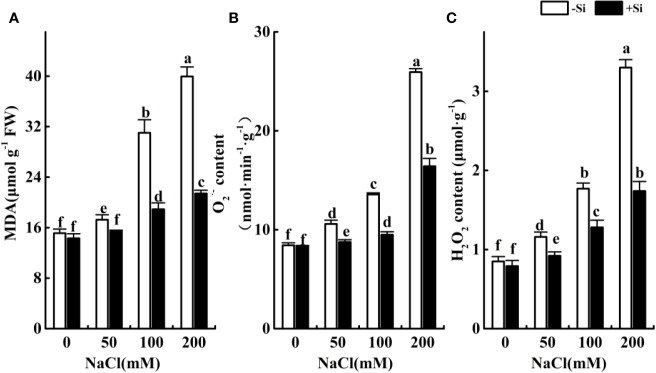
The effect of salt and Si treatment on MDA **(A)**, O_2_^•−^ **(B)** and H_2_O_2_ **(C)** content in alfalfa leaves. Four weeks-old alfalfa seedlings were exposed to 0, 50, 100, and 200 mM NaCl with (+2 mM Si) or without (−2 mM Si) Silicon for 14 d. Leaves were sampled 14 d after initiating salt treatment to determine MDA, O_2_^•−^ and H_2_O_2_ content. Bars indicate standard deviation (SD) of the means (*n* = 4). Different letters denote signiﬁcant differences among the treatments based on Duncan’s multiple range test (*P* < 0.05).

The contents of MDA, O_2_^•−^, and H_2_O_2_ significantly increased as the NaCl concentration increased from 0 to 200 mM (**Figures 4A–C**). However, the contents of MDA, O_2_^•−^ and H_2_O_2_ significantly increased due to Si supplementation under the NaCl concentration from 50 to 200 mM.

### Effect of Si on Photosynthesis Parameters in Leaves Under Salt Stress

**Figure 5:**
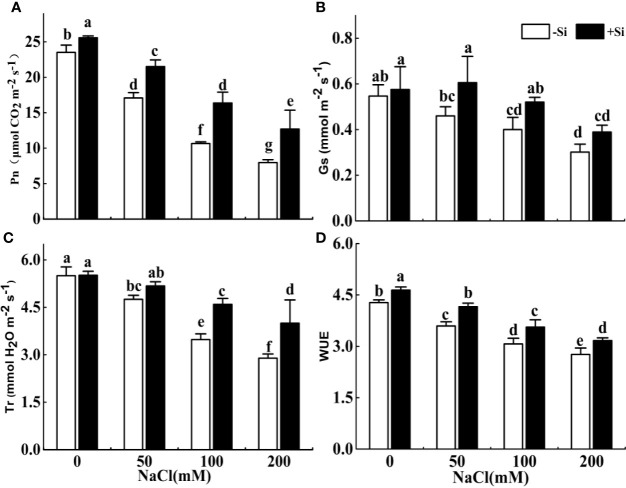
The effect of salt and Si treatment on the net photosynthesis rate (Pn) **(A)**, stomatal conductance (*gs*) **(B)**, transpiration rate (Tr) **(C)**, and water use efficiency (WUE) **(D)** in alfalfa leaves. Four weeks-old alfalfa seedlings were exposed to 0, 50, 100, and 200 mM NaCl with (+2 mM Si) or without (−2 mM Si) Silicon for 14 d. Leaves were measured 14 d after initiating salt treatment for photosynthesis parameters. Bars indicate standard deviation (SD) of the means (*n* = 4). Different letters denote signiﬁcant differences among the treatments based on Duncan’s multiple range test (*P* < 0.05).

The photosynthetic rate, transpiration rate, and WUE significantly decreased with the rise of NaCl concentration from 0 to 200 mM (**Figures 5A–D**). The *gs* showed a decrease as the NaCl concentration increased, but no significant difference was found between the adjacent treatments (**Figure 5B**). However, the photosynthetic rate and WUE significantly increased due to Si supplementation under each NaCl treatment. The Gs significantly increased due to Si supplementation under 50 and 100 mM treatments, while the Tr significantly improved due to Si supplementation under 100 and 200 mM NaCl treatments.

### Effect of Si on the Antioxidant Enzymes Performance in Leaves During Salt Exposure

**Figure 6:**
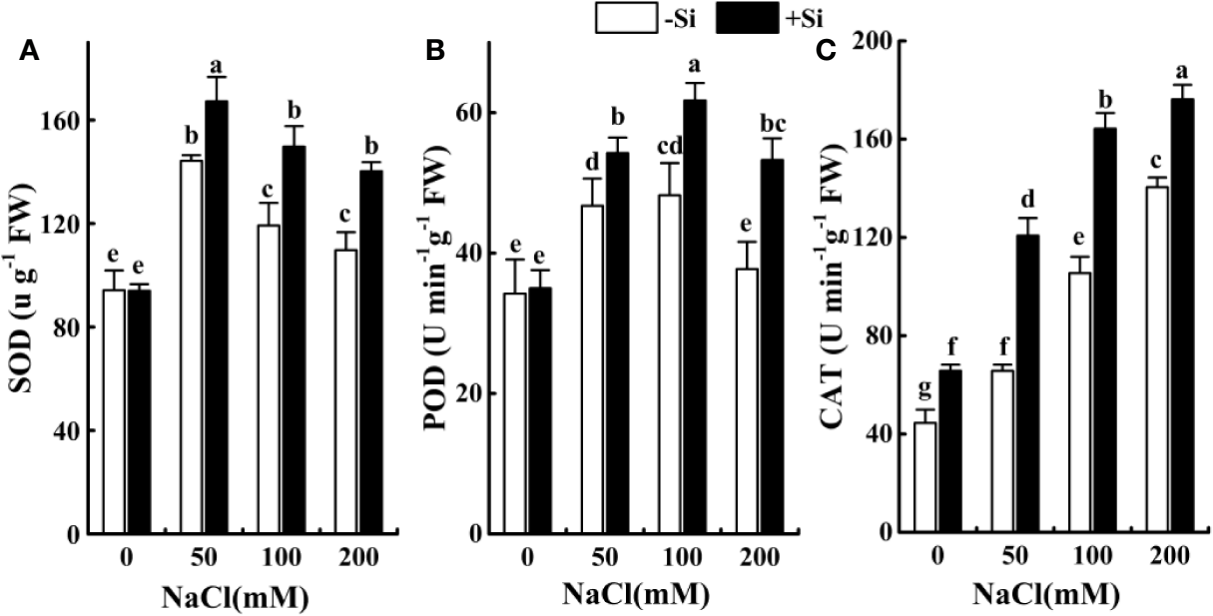
The effect of salt and Si treatment on the SOD **(A)**, POD **(B)** and CAT **(C)** in alfalfa leaves. Four weeks-old alfalfa seedlings were exposed to 0, 50, 100 and 200 mM NaCl with (+2 mM Si) or without (−2 mM Si) Silicon for 14 d. Leaves were sampled 14 d after initiating salt treatment for the determination of antioxidant enzymes. Bars indicate standard deviation (SD) of the means (*n* = 4). Different letters denote signiﬁcant differences among the treatments based on Duncan’s multiple range test (*P* < 0.05).

The activities of SOD initially increased at 50 mM NaCl treatment and then dropped at 100 and 200 mM NaCl treatment (**Figures 6A–C**). The POD activities followed the same trend, the maximum increase in NaCl-stressed alone alfalfa seedlings was observed at 50 mM NaCl treatment (**Figure 6B**), while that of CAT activities were recorded at 200 mM (**Figure 6C**). Si supplement increased the SOD, CAT, and POD activities in Si-treated NaCl-stressed alfalfa seedlings *versus* NaCl-stressed alone seedlings. The maximum increase in SOD and CAT activities in Si-treated NaCl-stressed plants was recorded at 100 mM NaCl, while that of POD was observed at 200 mM NaCl (**Figure 6A**). Under normal conditions, Si-treated seedlings showed higher activities of SOD, CAT, and POD *versus* control (**Figures 6A–C**).

## Discussion

In this study, alfalfa seedlings showed more tolerance to salt stress when 2 mM of Si solution was applied. This was evidenced by an enhanced shoot growth performance under salt stress conditions. We observed plant growth was significantly inhibited by NaCl stress. At the same time, the Si + NaCl-treated plants had higher stem extension rate, shoot DW, predawn LWP, and Chl contents under salt stress. Our data indicated that the exogenous application of Si significantly alleviated the damage of alfalfa seedlings under NaCl stress. This finding confirmed previous studies on wheat (Tuna et al., 2008), barley (Liang et al., 2006), soybean (Lee et al., 2010), canola (Haddad et al., 2018), and tomato (Romero-Aranda et al., 2006).

Results obtained in our experiment showed that salt stress damaged tissue structure and cell metabolism. This can be explained that Na^+^ reduced the performance of the antioxidant enzymes to cause ROS accumulation, resulting in lipid peroxidation. Na^+^ gradually accumulates in plant leaves with the increase of Na^+^ concentration in the soil. Our data exhibited that Si concentration was significantly increased with Si application, and the maximum recovery was observed at 100 mM NaCl. This is because higher Si concentration largely increased plant-available Si to protect the plant roots against sodium toxicity. Hurtado et al. (2019) reported that Si decreased Na^+^ content in sorghum and sunflower, especially when Si was root irrigated.

Salt stress severely inhibited plant growth and performance (Wei et al., 2017). Biomass is an important indicator of plant resistance (Zhang et al., 2017). Under NaCl stress, the shoot DW was significantly lower than that of non-NaCl stress. Moreover, the loss of shoot DW was greater than that of root DW. This finding was consistent with previous studies under salt stress (Khan et al., 1994; Debouba et al., 2006; Munns and Tester, 2008). Yin et al. (2013) which have shown that exogenous Si increased plant biomass by enhancing Pn under saline conditions. In this study, exogenous Si improved the growth performance of alfalfa seedlings under NaCl stress, especially the shoot DW. Similar observations were found in *Sorghum bicolor* with Si addition under salt stress (Yin et al., 2013). It has been known that exogenous Si can promote net photosynthesis under salt stress. The leaf anatomical structure, level of photosynthetic pigments, and activity of ribulose biphosphate carboxylase may be the reason for the improvement of silicon-mediated photosynthesis (Hamayun et al., 2010). In this study, exogenous Si increased total chlorophyll content under salt stress.

Salt stress interferes with the activity of photosynthesis due to osmotic stress, nutritional imbalance, and Na^+^ poisoning of photosynthetic organs (Rios et al., 2017). WUE, Tr, *gs*, and Pn of alfalfa seedlings were decreased under NaCl stress. However, these characteristics of alfalfa seedlings signiﬁcantly increased under the treatment of exogenous Si addition with NaCl stress. Similarly, Leaf gas exchange, WUE, and photosynthetic efficiency can be recovered by adding Si in a halophytic grass under high saline conditions (Mateos-Naranjo et al., 2013). Our results showed that exogenous Si enhanced all the photosynthesis parameters in alfalfa leaves under salt stress. In plants, maintenance of water homeostasis is an important response for plants to enhance salt resistance. Exogenous Si can enhance WUE through changing leaf keratin-bilayer structures or decreasing leaves transpiration losses (Guntzer et al., 2012; Shi et al., 2014; Li et al., 2015). However, our data showed exogenous Si increased transpiration rate under salt stress, indicating that Si-induced physical deposition was not the reason that plant WUE increased (Abdel Latef and Tran, 2016); In this study, exogenous Si increased WUE of alfalfa under salt stress, which was mainly due to exogenous Si improving photosynthesis of alfalfa.

The predawn LWP is a good indicator of the plant moisture status and can be used to present the soil water potential when transpiration doesn’t occur (Améglio et al., 1999). During stress, predawn LWP was a reliable indicator of alfalfa leaves’ water status (Hall and Larson, 1982). Maintaining good water status in cells and tissues can maintain metabolic activity through osmotic adjustments and other adaptations to salinity stress. In this study, osmotic stress was induced with the rise of NaCl concentration in the soil, which obstructed the root to absorb water, resulting in leaf water deficit and reduction in predawn LWP. de Oliveira et al. (2019) have shown that Si reduced water loss by decreasing the transpiration rate on sorghum plants. Si application significantly increased predawn LWP and Tr under salt stress. This indicated that Si application can improve the leaf water balance and alleviate salt-induced osmotic stress. It has been explained that Si-induced osmotic adjustment was the mechanism for the enhanced resistance of tomato plants under salt stress (Romero-Aranda et al., 2006). Sonobe et al. (2009) reported that Tr, root water uptake, and leaf water status were increased when Si was supplied to sorghum. Liu et al. (2015b) also showed that osmotic adjustment was the reason for Si-induced salt resistance, in which aquaporins participate in plant water response. The higher predawn LWP of alfalfa treated with Si under salt stress may be caused by the expression of certain aquaporins regulated by exogenous Si. Therefore, further research should be done to explore the effect of aquaporin expression on the Si-induced improvement on the water status of alfalfa under salt stress.

To date, there have been few reports related to the protective role of Si supplementation on the alfalfa oxidative defense system under saline stress. Studies have demonstrated that salt stress interfered with the production and removal of ROS, leading to the accumulation of more ROS that damages tissue structure and cell metabolism (Vaidyanathan et al., 2003; Zhu et al., 2015). Excess ROS causes lipid peroxidation and more accumulation of MDA (Shi et al., 2014; Zhang et al., 2017). In this study, the content of O_2_^•−^, H_2_O_2_, and MDA was accumulated as the NaCl concentration increased, which indicated ROS burst and NaCl-induced oxidative damage to alfalfa leaves. This result was confirmed by Xiong et al. (2018), who noted the MDA level of the two alfalfa cultivars increased dramatically with increasing NaCl level. Exogenous Si application significantly reduced the accumulation of O_2_^•−^ and H_2_O_2_ in alfalfa leaves under NaCl stress, indicating that Si triggered antioxidant enzyme performance to reduced ROS accumulation in NaCl-stressed seedlings. Further, exogenous Si application significantly increased the activities of SOD, CAT, and POD in alfalfa leaves under salt stress. These results demonstrated that under salt stress, Si improved the performance of the antioxidant enzymes and reduced the accumulation of ROS in leaves. Similar results have been conﬁrmed in barley and sorghum under salt conditions (Liang et al., 2003; Liu et al., 2015a).

In this study, salt stress resulted in an increase in leaf Na^+^ content and a decrease in leaf K^+^ content, causing a decrease in leaf K^+^/Na^+^ ratio. Cell-based mechanisms of ion homeostasis are essential determinants of salt stress adaptation (Munns and Tester, 2008). K^+^ is important for many biological processes in maintaining plant growth and stress tolerance. It has been known that maintaining a high K^+^/Na^+^ ratio would enhance plant resistance under salt stress (Anschütz et al., 2014; Himabindu et al., 2016). The decrease in K^+^ content under salt stress may be due to the inhibitory effect of Na^+^ toxicity and competition of Na^+^ and K^+^ ions for binding sites (Azooz et al., 2015). Si-treated alfalfa seedlings significantly reduced leaf Na^+^ content, while increased leaf K^+^ content, and thus maintained a higher leaf K^+^/Na^+^ ratio under NaCl stress. This reduction in Na^+^ content may be due to alfalfa root structure changes in the exodermis and endodermis by Si-deposition, which causes the decrease of the apoplastic Na+ absorption by roots under salt stress (Liu et al., 2015a). The increase of K^+^ content in wheat roots under water shortage could be that exogenous Si activated the plasma membrane H^+^-ATPases of the roots (Karmollachaab and Gharineh, 2015). At the same time, The high K^+^/Na^+^ ratio is also a good indicator related to exogenous Si on the absorption balance of K^+^ and Na^+^ under salt stress, which indicates that Si is an effective agricultural fertilizer element for alfalfa growing on saline soils.

## Conclusion

In summary, the exogenous application of Si can actively regulate leaf water balance and K^+^/Na^+^ homeostasis, reduced osmotic damage to leaves, and increased the Chl content, Pn, and shoot biomass of alfalfa under NaCl stress. In addition, exogenous Si enhanced antioxidant enzymes performance in leaves, decreased ROS accumulation and MDA content, thus improving the salt tolerance of alfalfa. Our results demonstrated that exogenous Si has potential application value in the cultivation of alfalfa in saline soils.

## Data Availability Statement

The datasets generated for this study are available on request to the corresponding author.

## Author Contributions

YM conceived and designed experiments. JZ and KF performed physiological and morphological measurements. YM and QY analyzed the data. YM wrote the manuscript and prepared the ﬁnal manuscript. QY, JN, and ZY supervised the experimental process. All authors contributed to the article and approved the submitted version.

## Funding

This study was financially supported by Ordos comprehensive experimental station of the technology system of pasture industry in China (CARS-34) and the 13th plan of 5 year national development of key research project of China (2016YFC0500605).

## Conflict of Interest

The authors declare that the research was conducted in the absence of any commercial or financial relationships that could be construed as a potential conflict of interest.
